# Ticks and Tick-Borne Pathogens: Occurrence and Host Associations over Four Years of Wildlife Surveillance in the Liguria Region (Northwest Italy)

**DOI:** 10.3390/ani14162377

**Published:** 2024-08-16

**Authors:** Lisa Guardone, Chiara Nogarol, Annalisa Accorsi, Nicoletta Vitale, Valeria Listorti, Sonia Scala, Sonia Brusadore, Ilaria Nina Miceli, Lara Wolfsgruber, Annalisa Guercio, Santina Di Bella, Francesca Grippi, Elisabetta Razzuoli, Maria Lucia Mandola

**Affiliations:** 1 S.S. Genova e Portualità, Istituto Zooprofilattico Sperimentale Piemonte, Liguria e Valle d’Aosta, Piazza Borgo Pila 39, 16129 Genova, Italy; annalisa.accorsi@izsto.it (A.A.); valeria.listorti@izsto.it (V.L.); lara.wolfsgruber@gmail.com (L.W.); 2 Department of Veterinary Sciences, University of Pisa, Viale delle Piagge 2, 56124 Pisa, Italy; 3 S.S. Virologia Specialistica, Istituto Zooprofilattico Sperimentale Piemonte, Liguria e Valle d’Aosta, Via Bologna 148, 10154 Turin, Italy; chiara.nogarol@izsto.it (C.N.); sonia.scala@izsto.it (S.S.); sonia.brusadore@izsto.it (S.B.); ilaria.miceli@izsto.it (I.N.M.); marialucia.mandola@izsto.it (M.L.M.); 4 S.S. Epidemiologia—Sanità Animale, Istituto Zooprofilattico Sperimentale Piemonte, Liguria e Valle d’Aosta, Via Bologna 148, 10154 Turin, Italy; nicoletta.vitale@izsto.it; 5 Centro Nazionale di Referenza per *Anaplasma*, *Babesia*, *Rickettsia* e *Theileria* (C.R.A.Ba.R.T.), Istituto Zooprofilattico Sperimentale della Sicilia A. Mirri, Via Gino Marinuzzi 3, 90129 Palermo, Italy; annalisa.guercio@izssicilia.it (A.G.); santina.dibella@izssicilia.it (S.D.B.); 6 S.C. Diagnostica Sierologica, Istituto Zooprofilattico Sperimentale della Sicilia A. Mirri, Via Gino Marinuzzi 3, 90129 Palermo, Italy; francesca.grippi@izssicilia.it

**Keywords:** vector-borne diseases, ticks, wildlife, wild boar, roe deer, fallow deer, *Rickettsia* spp.

## Abstract

**Simple Summary:**

Ticks are ectoparasites of animals and humans that can transmit pathogens usually referred to as Tick-borne pathogens (TBPs). This study analyzed 683 ticks collected from different wild animals (roe deer, wild boar, fallow deer, and chamois) hunted in Liguria between 2019 and 2022. Tick species were identified by morphological analysis; ticks from the same animal were grouped into homogeneous pools by species and life stage and submitted to molecular analysis for the detection of specific TBPs. Four different tick species were found: the most common was *Ixodes ricinus*, followed by *Dermacentor marginatus*, *Rhipicephalus sanguineus* s.s., and *Haemaphysalis punctata*. Almost 28% of the 222 tick pools analyzed were positive for at least one TBP. Most pools were positive for *Rickettsia* spp., and several species were found, all potential agents of human disease: *Rickettsia slovaca*, *R. monacensis*, *R. helvetica*, *R. massiliae*, and *R. raoultii*. Varying prevalences were found depending on the tick and host species. Other less frequent TBPs were *Anaplasma phagocytophilum* (three pools) and *B. burgdorferi* s.l. (one pool). All samples were negative for *Coxiella burnetii* and tick-borne encephalitis virus. Significant associations were found between *I. ricinus* and roe deer, *D. marginatus* and wild boar, and between *R. monacensis* and *I. ricinus.* The widespread presence of TBPs, particularly of several zoonotic *Rickettsia* species, requires the monitoring of domestic and wild animals and providing specific information to citizens for preventive actions.

**Abstract:**

Tick-borne diseases (TBDs) are a considerable public health problem worldwide. The occurrence of *Anaplasma* spp., *Borrelia burgdorferi* s.l., *Coxiella burnetii*, *Rickettsia* spp., and tick-borne encephalitis virus (TBEv) was investigated via PCR and sequencing in 683 ticks collected from 105 roe deer, 61 wild boars, 49 fallow deer, and 2 chamois, in the Liguria region, northwest Italy, between 2019 and 2022. The ticks were morphologically identified. Four different tick species were found: *Ixodes ricinus* (66.8% of the collected ticks), *Dermacentor marginatus* (15.8%), *Rhipicephalus sanguineus* s.s. (15.7%), and *Haemaphysalis punctata* (0.9%). Six ticks (0.9%) were only identified as *Rhipicephalus* spp. Of the 222 pools analyzed, 27.9% were positive. Most pools (n = 58, 26.1% of pools analyzed) were positive for *Rickettsia* spp., and several species were found: *Rickettsia slovaca* was the dominant species (15.3%), followed by *R. monacensis* (8.1%), while *R. helvetica* (1.8%), *R. massiliae* (0.5%), and *R. raoultii* (0.5%) were found only sporadically. *Anaplasma phagocytophilum* was identified in three pools and *B. burgdorferi* s.l. in one pool. All samples were negative for *C. burnetii* and TBEv. Significant associations were found between *I. ricinus* and roe deer, *D. marginatus* and wild boar, and between *R. monacensis* and *I. ricinus*. The prevalence of *Rickettsia* spp. differed significantly between tick and host species. This updated picture of tick species and TBPs in wild ungulates in Liguria, where the population of these animals is increasing, shows a widespread presence of potentially zoonotic *Rickettsia* spp. Continuous monitoring and public information on preventive measures are needed.

## 1. Introduction

Vector-borne diseases (VBDs) are a considerable public health issue worldwide. Interactions among pathogens, hosts, and environment play a key role in the emergence or re-emergence of VBDs. In addition, social and demographic factors such as human population growth, urbanization, globalization, trade exchanges, travel, and close interactions between livestock and wildlife have been significantly associated with the emergence and/or re-emergence of VBDs [1,2]. Indeed, an increase in both the incidence and the geographical range of VBDs has been noticed in recent decades, likely due to the expansion of the range of vectors associated with climate change [3].

Ticks are a major threat to human and animal health, as they are one of the most important arthropod vectors of pathogens to humans and wild and domestic animals [4]. Climate change is known to be closely related to the distribution and dynamics of tick populations by limiting biodiversity and favouring the survival of ticks, significantly increasing the development and density of these arthropods even at relatively high altitudes [4,5,6,7]. The most relevant zoonotic tick-borne pathogens (TBPs) responsible for VBDs include *Borrelia (B.) burgdorferi* sensu lato (s.l.), tick-borne encephalitis virus (TBEv), *Rickettsia* spp., *Anaplasma (A.) phagocytophilum*, and *Coxiella (C.) burnetii*. To date, Lyme borreliosis and TBE are the most prevalent VBDs in the northern hemisphere [7,8].

In Europe, the main vectors of borreliosis are hard ticks of the species *Ixodes (I.) ricinus* [7], which become infected when they feed on birds or mammals that carry the bacterium in their blood. Rodents and birds are reservoirs due to the competence of their immune systems, while the immune system of incompetent hosts, such as deer, can kill the bacteria. However, from an ecological point of view, wild ruminants are important for maintaining a large tick population in nature, as they are the preferred host species of adult ticks [7]. *Ixodes ricinus* is also one of the main vectors and reservoirs of the European subtype of TBEv, together with *I. persulcatus* [9]. Although ungulates are of particular interest due to their sentinel role in Flavivirus circulation and their indirect role in TBP maintenance as *Ixodes* feeders and spreaders [10,11], very few studies on TBEv have been conducted in Italy on wild ungulate and/or their feeding ticks. As regards *Rickettsia* species, a specificity for a single-tick host genus (or species) has been observed [12]. The transmission of *Rickettsia (R.) conorii*, the species believed to be the most frequently involved in human rickettsiosis in Europe so far, has historically been associated with the tick *Rhipicephalus (R.) sanguineus* [13], although this association is not so clear in the wild [14]. Another species, *R. slovaca*, is considered an emerging zoonotic species, and the main vector appears to be *Dermacentor* spp., particularly *Dermacentor (D.) marginatus*, which is the most common vector for the human transmission of this pathogen [15,16]. A broad spectrum of rickettsiae has also been detected in *I. ricinus*, the most widespread hard tick species in European countries. This tick appears to be a competent vector mainly for *R. helvetica* and *R. monacensis* [13]. Although human rickettsiosis caused by these two species is more rarely diagnosed, both have been reported in humans in different European countries, including in Italy [17,18]. *A. phagocytophilum* has been associated with *Ixodes* tick species in the northern hemisphere, including Europe, and wild ruminants may also be efficient reservoir hosts [19]. As for *C. burnetii*, which is responsible for reproductive disorders in domestic ruminants and a leading cause of abortion in sheep in Europe, its wild cycle is not fully known. It has been suggested that ticks act as a reservoir for *C. burnetii* and play a role in the transmission to vertebrate hosts in natural environments, mainly through tick feces and saliva, although this topic requires further studies [20,21,22,23,24].

The epidemiology of ticks is influenced by host-related and anthropogenic factors and impacts on the emergence and spread of TBPs. Knowledge of the type of tick species and TBPs present in a geographical area is essential for control strategies [25]. Wildlife has a major impact on tick epidemiology and is a useful tool for characterizing and monitoring tick populations and associated TBPs [26,27,28]. Wild ungulates are widespread across Europe, and several ungulate species have increased their densities and expanded their ranges in recent decades [29]. These changes in the wild ungulate ecosystem have allowed their ectoparasites, such as *I. ricinus*, to increase their density and expand their range [30], leading to an increased incidence of zoonotic TBPs.

The present survey aimed to investigate selected TBPs in ticks collected from wildlife in the Liguria region, by means of molecular analysis and sequencing. This will help to characterize the public health risk by providing useful data to better define the circulation of TBPs and their association with ticks/ungulate species in the regional territory, where data on these topics are scarce.

## 2. Materials and Methods

### 2.1. Tick Collection and Identification

Ticks were collected from wild animals from 2019 to 2022, as part of the regional plan for the monitoring and surveillance of wildlife in the Liguria region, northwest Italy, as previously described by Accorsi et al. [31]. Briefly, ears, tails, or parts of hides of hunted game, including wild boar (*Sus scrofa*), fallow deer (*Dama dama*), roe deer (*Capreolus capreolus*), and chamois (*Rupicapra rupicapra*), were delivered by hunting associations to the four local IZSPLV sections (Imperia, Savona, Genoa, La Spezia) of the Liguria region and stored at −20 °C until the analysis. It should be noted that the hunting season (defined by regional laws) varies for each species (see [31] for details), and therefore, sampling was performed throughout the year, but not continuously for each host species. Similarly, the sampling sites involved the whole region, but the sampling was opportunistic and not homogenously distributed over the territory. Ticks were carefully removed with forceps, stored at −20 °C (if needed) and examined via stereomicroscopy for morphological identification following Barker et al. [32], Estrada-Peña et al. [33], and Nava et al. [34]. Ticks collected from the same animal and belonging to the same species and life stage were pooled (mean n of ticks in a pool = 3, range 1–23), and stored at −20 °C until molecular analysis (Section 2.2).

### 2.2. Molecular Analysis for Pathogen Detection

#### 2.2.1. DNA/RNA Extraction

Tick homogenates were prepared from either (i) engorged female ticks (one tick was cut longitudinally into two equal parts using sterile forceps and surgical blades, and one half was used for nucleic acid extraction), (ii) a (sub-)pool of up to 4 non-engorged adults, or (iii) a (sub-)pool of up to 4 nymphs and/or larvae (grouped according to species, development stage, sex, and host). They were prepared by mechanical disruption using a Tissue Lyser (TissueLyser II, QIAgen, Hilden, Germany) and ceramic beads, followed by nucleic acid extraction using the Maxwell^®^ RSC viral TNA Kit procedure (Promega Corporation, Madison, WI, USA). Briefly, each sample was added directly to 200 µL of lysis buffer and 20 µL of proteinase K according to the manufacturer’s instructions. After the treatment with Tissue Lyser (30 Hz for 3 min), the homogenate was treated at 61 °C for 10 min, and after a centrifugation step, the entire volume was added to well #1 of the Maxwell^®^ RSC Cartridge according to the automated protocols of the Maxwell^®^ instrument (Promega Corporation, Madison, WI, USA). At the end of the procedure, 50–70 µL of total DNA/RNA extract was obtained.

#### 2.2.2. PCR Amplification for TBP Screening

The molecular analysis for the screening of TBPs targeting *Anaplasma* spp., *B. burgdorferi* s.l., *C. burnetii*, and *Rickettsia* spp. was performed using S.S. Virologia Specialistica using end-point PCR protocols in a final volume of 25 μL, using Platinum™ Taq DNA Polymerase (Invitrogen, Thermo Fisher Scientific Inc., Waltham, MA, USA) and 5 μL of each DNA extract. The primers, target genes, and references used for each PCR protocol are listed in Table 1. Positive controls (certified DNA from national reference laboratories) for each of the different pathogens were used in every assay; RNase-free water was used as the no-target control. Amplifications were carried out in a Thermal Cycler Applied Biosystem 2720. The amplicons were subjected to electrophoresis in a 1.5% of agarose gel and visualized using a Syber Safe nucleic acid staining solution under UV light. A 50–2000 bp DNA ladder was used as a molecular weight size marker (Amplisize^®^ Molecular Ruler, BioRad Laboratories, Hercules, CA, USA). PCR amplification for TBEv was carried out using the published method described by Schwaiger et al., [35], in a total volume of 20 μL using QuantiTect Multiplex PCR (QIAgen, Hilden, Germany) and 4 μL of RNA extract (Table 1).

All molecular analyses were conducted in S.S. Virologia Specialistica (Turin). All positive samples were then sent to the National Reference Centre for *Anaplasma*, *Babesia*, *Rickettsia* and *Theileria*—CRABART, Istituto Zooprofilattico Sperimentale Sicilia, Palermo, Italy.

#### 2.2.3. PCR Amplification for TBP Confirmation and Sequencing

The nucleic acids extracted from samples positive to the PCR amplification for TBP screening (Section 2.2.2) were sent to CRABART, where they were analyzed via end-point PCR, targeting the outer membrane protein A (OmpA) [40], outer membrane protein B (OmpB) [41], and citrate synthase (gltA) [39] genes to detect the presence of *Rickettsia* spp. DNA, and 16S-rRNA to detect *Anaplasma* spp. DNA [42] (Table 2). The PCR reactions were performed using GoTaq G2 DNA Polymerase (Promega Italia s.r.l., Milan, Italy) with 5 µL of each DNA extract in a final volume of 50 µL. *Rickettsia conorii* DNA (Amplirun, Vircell, Granada, Spain) and *A. phagocytophilum* DNA extracted from IFA slides (Fuller Laboratories, Fullerton, CA, USA) were used as positive controls. Nuclease-free water was used as a negative control. Amplicons were visualized by electrophoresis on a 2% agarose gel. The PCR products were quantified and sent to Macrogen Inc. (Macrogen Europe, Amsterdam, The Netherlands) for sequencing. The sequences obtained were analyzed using Bioedit software 7.7 (Tom Hall, Ibis Biosciences, Carlsbad, CA, USA) and compared for nucleotide sequence identity with reference strains in the GenBank database using the Basic Local Alignment Search Tool (BLAST) to identify species.

Specific real-time PCRs were carried out on the extracted nucleic acids to detect DNA from *C. burnetii*, targeting the IS1111 fragment [43], and *B. burgdorferi*, amplifying a fragment of the OspA region [44,45]. Both real-time PCRs were performed in a final volume of 20 µL, using 5 µL of extracted DNA, 10 µL of 2× SsoAdvanced Universal Probes Supermix (Biorad, Hercules, CA, USA), 250 nM primers, and 250 nM probe. Assays were performed on a Bio-Rad CFX96 system using the following thermal conditions: a hold step of 95 °C for 5 min, and 45 cycles of 95 °C for 15 s, and 60 °C for 30 s. *Coxiella burnetii* DNA (provided by Istituto Zooprofilattico Sperimentale delle Venezie, Legnaro, Italy) and *B. burgdorferi* s.l. DNA extracted from the IFA slides (Fuller Laboratories, Fullerton, CA, USA) were used as positive controls. Nuclease-free water was used as a negative control.

### 2.3. Database and Statistical Analysis

Data on collection date, hosts, geographical origin, tick species, stage, sex, and pathogens detected were organized in an Excel database and used for statistical analysis. The prevalence and 95% exact binomial confidence intervals (CIs) of tick species for each pathogen found in vertebrate hosts were calculated using a binomial exact test via SAS. Associations between tick, host, and pathogen were assessed using Pearson’s chi-squared test. A generalized linear model was used to estimate the probability of observing a pathogen given the presence of a host or infection with a tick species. For all statistical tests, a *p*-value < 0.05 was considered statistically significant. The software QGIS 3.34 was used to describe the pathogens/host/tick distribution.

## 3. Results

**Figure 1:**
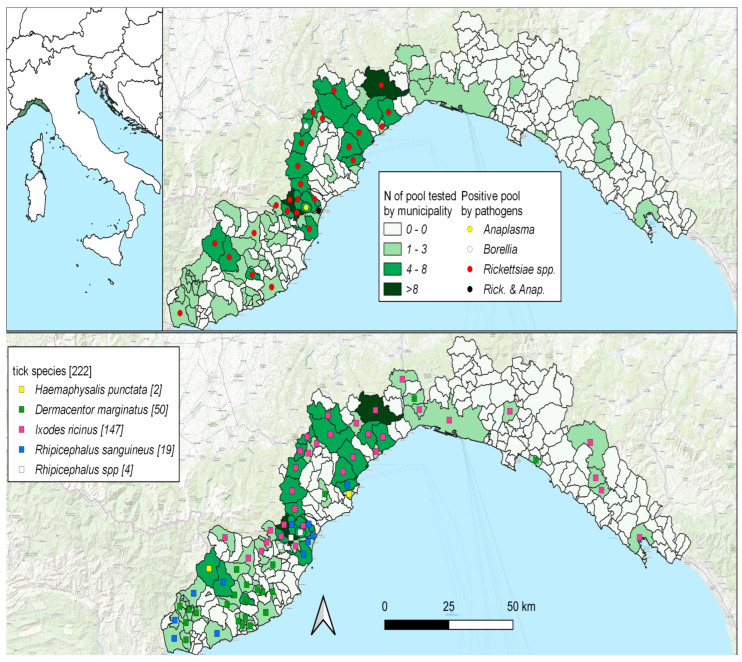
Distribution of tick species and positive pools by pathogens at municipality level.

A total of 683 ticks were collected from 217 wild animals (105 roe deer, 61 wild boar, 49 fallow deer, and 2 chamois), mainly from the western part of the region (Figure 1). Details of the tick species and the number of ticks collected for each host species are given in Table 3. Four different tick species were found: *Ixodes ricinus* (n = 456, 66.8% of the total collected ticks) was the most frequently collected species, followed by *Dermacentor marginatus* (n = 108, 15.8%), *Rhipicephalus sanguineus* s.s. (n = 107, 15.7%), and *Haemaphysalis punctata* (n = 6, 0.9%). Six ticks (0.9%) could only be identified to species level, such as *Rhipicephalus* spp.

From the collected ticks, 222 pools, consisting of 1–23 ticks, were created. Of these, 62 pools (27.9%) were positive for at least one of the pathogens tested at S.S. Virologia Specialistica. All the positive pools were sent to CRABART for confirmatory PCR and sequencing. The details of the results of the pathogen identification are reported in Table 4. All 56 pools were confirmed positive for *Rickettsia* spp. In addition, 2 pools that were only positive for *B. burgdorferi* s.l. were also positive for *Rickettsia* spp. during confirmatory testing, yielding a total of 58 positive pools for this pathogen. The dominant species was *R. slovaca*, found in 34 pools (54.8% of the positive pools), mainly from *I. ricinus*, but also from *D. marginatus* and *R. sanguineus*. The second most common species was *R. monacensis*, identified in 18 pools (29.0% of the positive pools). The other three species, *R. helvetica*, *R. massiliae*, and *R. raoultii*, were found sporadically, in four, one, and one pools, respectively.

The species *A. phagocytophilum* was confirmed in the three pools screened positive for *Anaplasma* spp.; interestingly, all three pools were also infected with *Rickettsia* spp. (in two cases with *R. monacensis* and in one case with *R. helvetica*). Concerning *B. burgdorferi* s.l., one pool was confirmed by CRABART, and a co-infection with *R. monacensis* was also found. Only one pool was positive for *C. burnetii* at the initial testing at the S.S. Virologia Specialistica laboratory, but this was not confirmed by CRABART. None of the pools tested positive for TBEv.

Table 5 describes the prevalence of TBPs in the analyzed pools according to the different hosts and tick species. *Rickettsia* spp. infected 30.61% of the *I. ricinus* pools (45 positive pools out of 147 pools tested; 95% CI = 23.28–38.74). The *I. ricinus* infection was higher (Pearson’s Chi squared test: 9.7, *p* > 0.002) in roe deer, with an overall prevalence of 34.96% (36/106; 95% CI = 25.82–44.98), compared to fallow deer, with a prevalence of 25% (9/36; 95% CI = 12.12–42.20). *Rickettsia* spp. was also found in 16% of *D. marginatus* tick pools (8/50; 95% CI = 7.17–29.11), and this tick species was found only in wild boars. Finally, *Rickettsia* spp. also infected *Rhipicephalus* spp. with a prevalence of 13.04% (3/23; 95% CI = 4.54–32.13), with the positive pools collected from one roe deer and two fallow deer.

Regarding the various *Rickettsia* species, most of the positive pools were composed of *I. ricinus* collected from roe deer, the only exception being a pool from a wild boar that tested positive for *R. raoultii*. A statistically significant association was observed between *R. monacensis* and *I. ricinus* (Fisher’s exact test *p* value = 0.0043; Chi squared test: 6.98, *p* value = 0.0083), and the probability to observe *R. monacensis* in *I. ricinus* was seven times higher than in the other tick species (OR 9.68; 95% CI 1.26–74.19). No significant associations were found between the other *Rickettsia* species and tick species.

The prevalence of *Rickettsia* spp. differed significantly between tick species (Pearson’s Chi squared test: 6.28, *p* > 0.05), with *I. ricinus* showing a higher prevalence than *D. marginatus* and *Rhipicephalus* spp. Other statistically significant factors were the following: host (Pearson’s Chi squared: test 10.8, *p* > 0.01), seasonality (Pearson’s Chi squared test: 25.66 *p* < 0.0001), and province (Pearson’s Chi squared test: 10.84 *p* < 0.0001). The higher prevalence was observed in 2020 in winter on the west side (Table 6, Figure 1).

Table 7 shows the results of the final logistic regression model (likelihood ratio χ^2^ = 40.25, degrees of freedom = 4, *p* < 0.001). Factors associated with *Rickettsia* spp. infection were the following: winter season, which showed a 13.6 times higher probability of observing a positive pool (OR 13.6; 95% CI 3.1–59.7); and host, as in roe deer, the probability to observe a positive pool was almost 3.4 (OR 2.3; 95% CI 1.6–7.1) when compared with others hosts. Roe deer was strongly associated with *I. ricinus*, so *I. ricinus* was removed from the final model. No association was observed between the positivity for *A. phagocytophilum*. and *B. burgdorferi* s.l. with the considered factors (host, tick species, season, area).

## 4. Discussion

It is known that wild ungulates play a central role in the life cycle of ticks [46] and that they may play an important role in the ecology of TBPs, acting as maintenance hosts for tick populations and, in some cases, as natural reservoirs of some TBPs [47,48]. The finding of *I. ricinus* as the most abundant species, and on a large range of vertebrate hosts, agrees with most studies investigating the occurrence of ticks in Italy and in Europe [49,50,51,52,53,54,55]. Climate change, habitat fragmentation, and the numerical increase in vertebrate hosts even in new habitats have increased the density and geographical range of *I. ricinus* [56,57]. The remaining ticks found in this study were almost equally represented by *D. marginatus* and *R. sanguineus* s.s. However, while *D. marginatus* was only found on wild boar, supporting this tick–host association [31,58], *R. sanguineus* s.s. was found on all vertebrate hosts: roe deer, fallow deer, chamois, and wild boar. When comparing our results with those of other studies conducted in Italy and Europe, it is important to consider that sampling methods and timeframe may differ. For instance, our study only relied on ticks collected from dead animals sampled during hunting seasons (which are variable for each animal species, as described by Accorsi et al. [31]), whereas Pascucci et al. [49] mainly collected free-living ticks via dragging in spring. This last sampling technique and season were considered the most suitable for *I. ricinus* due to its hunting behaviour (ambushing) and seasonal dynamics. Moreover, the technique may influence the life stage of the ticks collected: the collection of a high number of immature stages via dragging has been reported for *I. ricinus* [49], while no immature stages of *D. marginatus* have been collected via dragging in other surveys [50], thus suggesting a different questing behaviour of larvae and nymphs in the different tick species.

However, regardless of the technique used, as mentioned above, *I. ricinus* seems to be the most abundant species in Europe [49,50,51,59,60,61], and several studies agree that the species is expanding its geographical range, both by colonizing new areas and by expanding at higher altitudes [52,58,62]. In Italy, *I. ricinus* is mainly associated with the wooded areas of the northeastern and northwestern regions, where this species finds optimal conditions for its development in terms of temperature (i.e., 20–23 °C) and relative humidity (i.e., 85–98%) [63]. In addition, the species has been increasingly reported in urban green areas, where the likelihood of tick bites to humans and pets can be high [64,65]. In a survey conducted in the South of France, an area geographically close to the region studied in the present study, *I. ricinus* was found as the dominant species even in sites and landscapes where its presence had not been previously reported. Indeed, *I. ricinus* is very sensitive to desiccation and is generally considered to be absent from dry landscapes, such as the Mediterranean coast [52]. The authors hypothesized that the geology of the Alpes–Maritimes region, consisting of several valleys with partial exposure to sunlight, may create suitable habitats for hygrophilic ticks such as *I. ricinus*. These geological features may act as fresh and humid ecological niches for this species, along with a Mediterranean climate and the presence of suitable hosts such as wild ruminants that can carry ticks [52]. All these considerations can be applied to the adjacent region of Liguria, with a similar orography and an analogue abundance of wild ruminants [31]. In fact, a widespread presence of *I. ricinus* in Liguria has already been observed by Ceballos et al. [66].

*Dermacentor marginatus* was only collected from wild boars in this study. This strong tick–host association agrees with previous studies [50,59]. Indeed, this tick was found as the most abundant species associated with wild boar populations in several areas of the Mediterranean basin, such as northeastern Spain [67], Corsica [68], Liguria and Sardinia [50], and southern Italy [59].

*Rhipicephalus sanguineus* develops at higher temperatures (e.g., 20–35 °C) and variable relative humidity (e.g., 35–95%) compared to other ticks, such as *I. ricinus* [69]. This tick species has a close evolutionary relationship with domestic dogs, which are its main hosts, but it has been reported from a wide range of ecological niches and many wild and domestic species, including humans [69,70]. Indeed, in the present survey, *R. sanguineus* s.s. was collected from fallow deer, roe deer, and wild boar.

*Haemaphysalis punctata* was found occasionally in this study, while it was the dominant species in Monti Sibillini National Park (central Italy), where almost all (98.9%) the specimens collected via dragging belonged to this species [71]. In the present study, five out of the six *H. punctata* collected were found on a chamois. *H. punctata* was reported from the southern chamois (*Rupicapra pyrenaica*) in Spain [72], while in another study [73] conducted in France, this tick species was found only in muflons and not in chamois.

Regarding pathogens, *Rickettsia* spp. was the most widespread genus. The prevalence of *Rickettsia* spp. found in this study is comparable to that found by Ebani and collaborators [74] on hunted wild animals in Tuscany (20.78% of the analyzed pools), but significantly lower than that reported in a similar study carried out on ticks collected from wildlife in the Abruzzo region (52.25%) [75]. Different ways of pooling ticks in the published studies may have affected the pathogen prevalence values, making them not easily comparable [49]. The sequencing analysis of *Rickettsia* spp.-positive pools identified five different species. *Rickettsia slovaca* was the most abundant, followed by *R. monacensis*, while *R. helvetica*, *R. massiliae*, and *R. raoultii* appeared to be less widespread. A diversity of *Rickettsia* spp. was already found in several studies [48,49,73,76,77,78]. Indeed, thanks to the improved diagnostic skills and, mainly, to the use of molecular tools, several spotted fever group (SFG) rickettsiae have been detected in the Italian territory in the last decade, including *R. slovaca*, *R. aeschlimanni*, *R. massilliae*, *R. monacensis*, *R. conorii* subsp. *israelensis*, *R. conorii* subsp. *indica*, *R. raoultii*, *R. helvetica*, *R. hoogstraalii*, *R. peacockii*, *R. rhiphicephali*, and *R. felis* [49].

Tick-borne SFG rickettsiosis is currently considered endemic in Italy. Although *R. conorii* was previously thought to be the only species responsible for human rickettsiosis in this country, various other species, such as *R. slovaca*, *R. monacensis*, *R. massiliae*, and *R. aeschlimannii* have recently been associated with human disease [10,18,79,80,81,82,83]. A report on the epidemiology of rickettsioses in the European Union/European Free Trade Association (EU/EFTA) countries was published in 2013 by the European Centre for Disease Prevention and Control (ECDC), describing the recognized *Rickettsia* species causing disease in humans, the specific illness, and the geographical distribution [84]. More recently, in a systematic review and modelling analysis, Zhang et al. [85] offered a comprehensive and up-to-date picture of 17 major SFG species, mapping global distributions and predicted risks in animals, vectors, and humans. The authors concluded that the wide spectrum of vectors contributes significantly to the increasing incidence of SFG infections among humans and that the potential risk areas are more extensive than previously reported. These findings underline the need for additional awareness, diagnosis, and surveillance.

Among these emerging species, *R. slovaca* has gained increasing importance [81,86]. In the present survey, *R. slovaca* was detected only in six pools of *D. marginatus* from wild boar and in three pools of *R. sanguineus* s.s. from roe deer and fallow deer, while it was mainly identified in pools of *I. ricinus* from the same ungulate species. This result does not agree with the literature data, where *R. slovaca* is reported to be frequently associated with *Dermacentor* spp. [86,87]. For example, Grassi et al. [86] carried out a study in the Euganean Hills Regional Park (northeastern Italy) on ticks sampled from animals and from the vegetation using the dragging method: they found a higher prevalence of *R. slovaca* in *D. marginatus* than in *I. ricinus*. Moreover, in the experimental study by Boldis et al., [87], a quantitative real-time PCR was used to characterize the growth of *R. slovaca* strain B in static (cell lines) and dynamic (*D. marginatus* and *I. ricinus* ticks) culture systems, showing that *D. marginatus* seems to be a more suitable environment for *R. slovaca* than *I. ricinus*. Accordingly, this pathogen was found with a remarkable prevalence in host-seeking *D. marginatus* along the Tyrrhenian coastline and from the Western Alps [88,89], and it was also found in *D. marginatus* ticks in Sardinia [76]. The *R. slovaca*/*D. marginatus* association was also found in ticks from humans by Blanda et al. [90] in Sicily. In humans, the most frequent clinical manifestation of *R. slovaca* is a syndrome characterized by scalp eschars and neck lymphadenopathy following the tick bite. The term SENLAT (scalp eschar and neck lymphadenopathy after a tick bite) was proposed in 2010 for this clinical entity: to date, the former and still-used names are TIBOLA (tick-borne lymphadenopathy) and DEBONEL (Dermacentor-borne necrotic erythema and lymphadenopathy) [91]. The disease is common in southern Europe, in parts of central Europe, and in central Asia, and European human cases have been described mainly in Spain, France, Hungary, Poland, and Portugal [13,92]. In Italy, only six microbiologically confirmed cases of SENLAT have been reported in Italy between 1996 and 2021. In these cases, *R. slovaca* and *R. massiliae* were identified as the causative agents through molecular methods. Ten additional SENLAT cases were reported from Tuscany between 2015 and 2022 [82], while northeastern Italy has been poorly investigated, not only for *R. slovaca*, but for the occurrence of rickettsiosis in general [81].

The second most frequent *Rickettsia* spp., *R. monacensis*, was mainly associated with *I. ricinus*, as already observed in Italy [49] and central Europe [92]. In this study, it was found in pools from roe deer and fallow deer. A pool of *D. marginatus* from a wild boar also tested positive, confirming the involvement of tick species other than *I. ricinus* in its transmission, as reported in several Italian regions and central Europe [49,75,92,93,94,95]. On the contrary, *R. helvetica* was identified exclusively in ticks of *I. ricinus* species collected from roe deer and fallow deer, supporting the role of this tick species as the main vector and natural reservoir [49,91]. Both *R. monacensis* and *R. helvetica* are recognized as occasional agents of spotted fever in Italy [18,93] and other countries [13].

In our study, *R. massiliae*, another potential agent of human disease [96], was only detected in one pool of *I. ricinus*, unlike other studies that reported its presence mainly in *Rhipicephalus* ticks [49,75,76,91]. *Rickettsia raoultii* was found in one pool of *D. marginatus*, as previously reported in Sardinia [76]. Indeed, this species was observed to be associated with *Dermacentor* ticks in Europe and Russia since its first description [97].

*Anaplasma phagocytophilum* was found in only three pools of *I. ricinus* from fallow deer. On the contrary, in a study conducted in Tuscany, an area of central Italy considered to be endemic for this pathogen, it was found in 29.87% of the tested pools [74]. The obtained results confirm both the role of *I. ricinus* as one of the main vectors of this pathogen in Europe and of wild ruminants as reservoirs [19]. Specifically, high roe deer densities were associated with high tick densities: these two parameters seem to have a positive effect on the *A. phagocytophilum* prevalence. In Europe, roe deer show prevalence rates up to 98.9%, but other deer species such as red deer and fallow deer may also be efficient reservoir hosts [19]. Moreover, A. *phagocytophilum* was found also in wild boar [59]. In 1994, *A. phagocytophilum* was found to cause disease in humans and identified as the causative agent of what was later named human granulocytic anaplasmosis (HGA) [9]. *Anaplasma phagocytophilum* is the main species associated with HGA [18]. However, zoonotic infections by *A. ovis* [98], and, more recently, by *A. caprae* [99] and *A. bovis* [100,101], have occasionally been reported. In Italy, cases of HGA were diagnosed in the northeast of the country, Sardinia and Sicily [18].

The only positivity for *B. burgdorferi* s.l. was observed in a pool of *I. ricinus* from a roe deer. Despite the apparently low occurrence of the pathogen in our study area, this TBP should not be disregarded, as the number of reported cases of Lyme borreliosis in Europe has increased significantly in recent years [52].

Finally, TBEv was not found in the pools analyzed in this study: this result is consistent with the epidemiological situation in the north of Italy, as the virus appears to be currently present only in the northeastern part of the country [102].

## 5. Conclusions

To date, ungulate management represents a tool that can be used to mitigate the risk of zoonotic diseases, and different ungulate species may play a role in the tick spread and transmission cycles of TBPs [65], so it appears strategic and necessary to study the potential role of wildlife as hosts of TBPs. Indeed, each pathogen may interact differently with different host species: targeted control measures on the specific ungulate species could influence the abundance and/or disappearance of infected ticks in some areas. To date, it is unclear whether and how different ungulate species differ in terms of their relative contribution to the tick life cycle and the transmission of tick-borne pathogens. Although numerous studies have investigated the role of ungulates in the transmission of TBPs, few have examined multiple ungulate species simultaneously [103,104], as in the present study. In particular, data on wild boar and fallow deer need to be expanded [64]. Data on wild boar are especially needed considering that the density of this species has increased throughout Europe, with high abundance in several countries, including Italy. In the investigated area, the density is particularly high, and wild boars are often found in urban and peri-urban areas, possibly contributing to the maintenance of ticks in such environments, favouring human exposure. Thus, continuous monitoring and information for citizens on preventive measures are needed.

## Figures and Tables

**Table 1:** Primers, target genes, and references for the PCR protocols used in the screening of tick-borne pathogens (TBPs).

Pathogen	Primers	Target Gene	Reference
*Anaplasma* spp.	16SANA-F 5′-CAGAGTTTGATCCTGGCTCAGAACG-3′ 16SANA-R 5′-GAGTTTGCCGGGACTTCTTCT GTA-3′	16S rRNA	Stuen et al. [36]
*Borrelia burgdorferi* s.l.	FLA1 5′-AGAGCAACTTACAGACGAAATTAAT-3′ FLA2 5′-CAAGTCTATTTTGGAAAGCACCTAA-3′	FLA	Skotarczak et al. [37]
*Coxiella burnetii*	Trans1 5′-TATGTATCCACCGTAGCCAG C-3′ Trans2 5′-CCCAACAACACCTCCTTATTC-3′	IS1111	Berri et al. [38]
*Rickettsia* spp.	RpCS.877p 5′-GGGGGCCTGCTCACGGCGG-3′ RpCS.1258n 5′-ATTGCAAAAAGTACAGTGAACA-3′	citrate synthase	Regnery et al. [39]
Tick-borne encephalitis (TBE) virus	F-TBE 5′-GGGCGGTTCTTGTTCTCC-3′ R-TBE 5′-ACACATCACCTCCTTGTCAGACT-3′ TBE-Probe-WTTGAGCCACCATCACCCAGACACA	3′ non-coding region	Schwaiger et al. [35]

**Table 2:** Primers, target genes, and references for PCRs performed to amplify different *Rickettsia* spp. molecular targets and *Anaplasma* spp. and real-time PCRs performed to amplify *Coxiella burnetii* and *Borrelia burgdorferi* s.l. for confirmation of tick-borne pathogens (TBPs).

Pathogen	Primers	Target Gene	Reference
*Rickettsia* spp.	Rr190.70p 5′-ATGGCGAATATTTCTCCAAAA-3′ Rr190.701n 5′-GTTCCGTTAATGGCAGCATCT-3′ Rr190.602n 5′-AGTGCAGCATTCGCTCCCCCT-3′	OmpA	Oteo et al. [40]
	rompB OF 5′-GTAACCGGAAGTAATCGTTTCGTAA-3′ rompB OR 5′-GCTTTATAACCAGCTAAACCACC-3′ rompB SFG IF 5′-GTTTAATACGTGCTGCTAACCAA-3′ rompB SFG IR 5′-GGTTTGGCCCATATACCATAAG-3′	OmpB	Choi et al. [41]
	RpCS.877p 5′-GGGGGCCTGCTCACGGCGG-3′ RpCS.1258n 5′-ATTGCAAAAAGTACAGTGAACA-3′	Citrate synthase	Regnery et al. [39]
*Anaplasma* spp.	EE1 5′-TCCTGGCTCAGAACGAACGCTGGCGGC-3′ EE2 5′-AGTCACTGACCCAACCTTAAATGGCTG-3′ EE3 5′-GTCGAACGGATTATTCTTTATAGCTTGC-3′ EE4 5′-CCCTTCCGTTAAGAAGGATCTAATCTCC-3′	16S-rRNA	Richter et al. [42]
*Coxiella burnetii*	sIS1pri F 5′-CGGGTTAAGCGTGCTCAGTAT-3′ sIS1pri R 5′-TCCACACGCTTCCATCACCAC-3′ Tqpro sIS1 (5′-FAM/3′-BHQ1) 5′-AGCCCACCTTAAGACTGGCTACGGTGGAT-3′	IS1111	Schets et al. [43]
*Borrelia burgdorferi* s.l.	Bor_OspA_F 5′-AATATTTATTGGGAATAGGTCTAA-3′ Bor_OspA_R 5′-CACCAGGCAAATCTACTGA-3′ Bor_OspA_TM (5′-FAM/3′-BHQ1) 5′-TTAATAGCATGYAAGCAAAATGTTAGCA-3′	OspA	Briciu et al. [44]

**Table 3:** The numbers of ticks collected for each host species, with the percentage of each tick species over the total number of ticks collected for that host species. The associations between hosts and ticks assessed by Fisher’s exact test are also shown. Statistically significant associations are indicated by *. n = number of hosts examined; ns = not significant associations.

Identified Tick Species	Roe Deer (n Host = 105)	Fallow Deer (n Host = 49)	Chamois (n Host = 2)	Wild Boar (n Host = 61)	Overall
*Ixodes ricinus*	276 (91.1%) ***	168 (74.7%) **	0 ***	12 (8.2%) ***	456 (66.8%)
*Dermacentor* *marginatus*	0 ***	0 ***	0 ^ns^	108 (73.5%) ***	108 (15.8%)
*Rhipicephalus* *sanguineus* s.s.	24 (7.9%) ***	53 (23.6%) ***	3 (37.5%) ^ns^	27 (18.4%) ^ns^	107 (15.7%)
*Rhipicephalus* spp.	3 (1.0%) ^ns^	3 (1.3%) ^ns^	0 ^ns^	0 ^ns^	6 (0.9%)
*Haemaphysalis* *punctata*	0 *	1 (0.4%) ^ns^	5 (62.5%) ***	0 ^ns^	6 (0.9%)
Overall number of collected ticks	303	225	8	147	683

^ns^ *p* > 0.05, * *p* ≤ 0.05, ** *p* < 0.001, *** *p* < 0.0001.

**Table 4:** Details of the identified pathogens in relation to tick and host species and number of pools confirmed positive.

Identified Pathogen	Tick Species	Host Species	PCR and Sequencing (CRABART): N of Confirmed Positive Pools
*Rickettsia slovaca*	*Ixodes ricinus*	Roe deer	20
		Fallow deer	5
	*Dermacentor marginatus*	Wild boar	6
	*Rhipicephalus sanguineus*	Fallow deer	2
		Roe deer	1
*Rickettsia monacensis*	*Ixodes ricinus*	Roe deer	13
		Fallow deer	4
	*Dermacentor marginatus*	Wild boar	1
*Rickettsia helvetica*	*Ixodes ricinus*	Roe deer	3
		Fallow deer	1
*Rickettsia massiliae*	*Ixodes ricinus*	Roe deer	1
*Rickettsia raoultii*	*Dermacentor marginatus*	Wild boar	1
*Anaplasma phagocytophilum*	*Ixodes ricinus*	Fallow deer	3
*B. burgdorferi* s.l.	*Ixodes ricinus*	Roe deer	1
*Coxiella burnetii*	*Ixodes ricinus*	Roe deer	0

**Table 5:** Prevalence of tick-borne pathogens (TBPs) in analyzed pools according to different hosts and tick species.

Host	Tick Species	N Ticks	Tested Pools	*Rickettsia slovaca* % [95% CI]	*Rickettsia monacensis* % [95% CI]	*Rickettsia helvetica* % [95% CI]	*Rickettsia massiliae* % [95% CI]	*Rickettsia raoultii* % [95% CI]	*Anaplasma* sp. % [95% CI]	*Borrellia* sp. [95% CI]
Chamois	*Haemaphysalis punctata*	5	1	0	0	0	0	0	0	0
Chamois	*Rhipicephalus sanguineus* s.s.	3	1	0	0	0	0	0	0	0
Roe deer	*Ixodes ricinus*	263	103	19.42 [12.28–28.38]	11.65 [6.17–19.47]	2.91 [0.60–8.28]	0.97 [0.02–5.29]	0	0	0.97 [0.02–5.29]
Roe deer	*Rhipicephalus sanguineus* s.s	24	3	33.33 [0.84–90.57]	0	0	0	0	0	0
Roe deer	*Rhipicephalus* spp.	3	1	0	0	0	0	0	0	0
Wild boar	*Dermacentor marginatus*	106	50	12.00 [4.53–24.31]	2.00 [0.05–13.65]	0	0	2.00 [0.05–13.65]	0	0
Wild boar	*Ixodes ricinus*	12	8	0	0	0	0	0	0	0
Wild boar	*Rhipicephalus sanguineus* s.s.	28	3	0	0	0	0	0	0	0
Fallow deer	*Haemaphysalis punctata*	1	1	0	0	0	0	0	0	0
Fallow deer	*Ixodes ricinus*	167	36	13.89 [4.67–29.50]	8.33 [1.75–22.47]	2.78 [0.07–14.53]	0	0	8.33 [1.75–22.47]	0
Fallow deer	*Rhipicephalus sanguineus* s.s.	51	12	8.33 [0.21–38.48]	0	0	0	0	0	0
Fallow deer	*Rhipicephalus* spp.	5	3	33.33 [0.84–90.57]	0	0	0	0	0	0

**Table 6:** Univariate analysis of factors associated with *Rickettsia* spp. infection in 222 pooled samples of ticks.

Factors	Category	n° Pool	*Rickettsia* spp. Prevalence	Chi Square	*p* Value
Host	Chamois	2	0.00%	10.83	0.01
	Roe deer	107	34.58%		
	Wild boar	61	13.11%		
	Fallow deer	52	21.15%		
Tick species	*Dermacentor marginatus*	50	12.73%	6.28	0.04
	*Ixodex ricinus*	147	37.42%		
	*Rhipicephalus* spp.	23	5.85%		
Season	Autumn	37	0.00%	25.67	<0.0001
	Spring	30	7.69%		
	Winter	155	34.84%		
Area	East	12	0.00%	4.28	0.04
	West	210	26.67%		

**Table 7:** Final multivariate logistic regression model for the presence of *Rickettsia* in pool of ticks. For each parameter Odds ratio (OR) 95% CI OR, the Wald χ^2^ and *p* values are provided.

Parameter	Baseline	OR	95% CI OR	Wald χ^2^	*p*-Value χ^2^
host	Roe deer	3.2	1.4–7.4	10.1384	0.0015
season	winter	12.9	2.9–57.0	11.9671	0.0005

## Data Availability

The original contributions presented in this study are included in the article; further inquiries can be directed to the corresponding authors.

## References

[1] Perry B.D., Grace D., Sones K. (2011). Current drivers and future directions of global livestock disease dynamics. Proc. Natl. Acad. Sci. USA.

[2] Tong M.X., Hansen A., Hanson-Easey S., Cameron S., Xiang J., Liu Q., Sun Y., Weinstein P., Han G.S., Williams C. (2015). Infectious diseases, urbanization and climate change: Challenges in future China. Int. J. Environ. Res. Public Health.

[3] Ebani V., Cerri D., Fratini F., Ampola M., Andreani E. (2008). Seroprevalence of *Anaplasma phagocytophilum* in domestic and wild animals from central Italy. New Microbiol..

[4] Gilbert L. (2021). The Impacts of Climate Change on Ticks and Tick-Borne Disease Risk. Annu. Rev. Entomol..

[5] IPCC (2014). Climate Change 2014: Impacts, Adaptation, and Vulnerability.

[6] Keesing F., Belden L.K., Daszak P., Dobson A., Harvell C.D., Holt R.D., Hudson P., Jolles A., Jones K.E., Mitchell C.E. (2010). Impacts of biodiversity on the emergence and transmission of infectious diseases. Nature.

[7] Boulanger N., Boyer P., Talagrand-Reboul E., Hansmann Y. (2019). Ticks and tick-borne diseases. Med. Mal. Infect..

[8] Stroffolini G., Segala F.V., Lupia T., Faraoni S., Rossi L., Tomassone L., Zanet S., De Rosa F.G., Di Perri G., Calcagno A. (2021). Serology for *Borrelia* spp. In Northwest Italy: A Climate-Matched 10-Year Trend. Life.

[9] Heyman P., Cochez C., Hofhuis A., Van Der Giessen J., Sprong H., Porter S.R., Losson B., Saegerman C., Donoso-Mantke O., Niedrig M. (2010). A clear and present danger: Tick-borne diseases in Europe. Expert Rev. Anti-Infect. Ther..

[10] Grassi L., Drigo M., Zelená H., Pasotto D., Cassini R., Mondin A., Franzo G., Tucciarone C.M., Ossola M., Vidorin E. (2023). Wild ungulates as sentinels of flaviviruses and tick-borne zoonotic pathogen circulation: An Italian perspective. BMC Vet. Res..

[11] Garcia-Vozmediano A., Bellato A., Rossi L., Hoogerwerf M.N., Sprong H., Tomassone L. (2022). Use of Wild Ungulates as Sentinels of TBEV Circulation in a Naïve Area of the Northwestern Alps, Italy. Life.

[12] Perlman S.J., Hunter M.S., Zchori-Fein E. (2006). The emerging diversity of *Rickettsia*. Proc. R. Soc. B Boil Sci..

[13] Portillo A., Santibáñez S., García-Álvarez L., Palomar A.M., Oteo J.A. (2015). Rickettsioses in Europe. Microbes Infect..

[14] Socolovschi C., Gaudart J., Bitam I., Huynh T.P., Raoult D., Parola P. (2012). Why are there so few *Rickettsia conorii conorii*-infected *Rhipicephalus sanguineus* ticks in the wild?. PLoS Negl. Trop. Dis..

[15] Silva-Pinto A., De Lurdes Santos M., Sarmento A. (2014). Tick-borne lymphadenopathy, an emerging disease. Ticks Tick Borne Dis..

[16] Socolovschi C., Mediannikov O., Raoult D., Parola P. (2009). The relationship between spotted fever group rickettsiae and ixodid ticks. Vet. Res..

[17] Oteo J.A., Portillo A. (2012). Tick-borne rickettsioses in Europe. Ticks Tick Borne Dis..

[18] Guccione C., Colomba C., Tolomeo M., Trizzino M., Iaria C., Cascio A. (2021). Rickettsiales in Italy. Pathogens.

[19] Stuen S., Granquist E.G., Silaghi C. (2013). *Anaplasma phagocytophilum*—A wide spread multi-host pathogen with highly adaptive strategies. Front. Cell. Infect. Microbiol..

[20] Arricau-Bouvery N., Rodolakis A. (2005). Is Q fever an emerging or re-emerging zoonosis?. Vet. Res..

[21] Berri M., Souriau A., Crosby M., Crochet D., Lechopier P., Rodolakis A. (2001). Relationships between the shedding of *Coxiella burnetii*, clinical signs and serological responses of 34 sheep. Vet. Rec..

[22] Rodolakis A. (2006). Q fever State of art, epidemiology, diagnosis and prophylaxis. Small Rumin. Res..

[23] Muskens J., van Maanen C., Mars M.H. (2011). Dairy cows with metritis: *Coxiella burnetii* test results in uterine, blood and bulk milk samples. Vet. Microbiol..

[24] Agerholm J.S. (2013). *Coxiella burnetii* associated reproductive disorders in domestic animals- a critical review. Acta Vet. Scand..

[25] Estrada-Peña A., Roura X., Sainz A., Miró G., Solano-Gallego L. (2017). Species of ticks and carried pathogens in owned dogs in Spain: Results of a one-year national survey. Ticks Tick-Borne Dis..

[26] del Cerro A., Oleaga A., Somoano A., Barandika J.F., García-Pérez A.L., Espí A. (2022). Molecular identification of tick-borne pathogens (*Rickettsia* spp., *Anaplasma phagocytophilum*, *Borrelia burgdorferi sensu lato*, *Coxiella burnetii* and piroplasms) in questing and feeding hard ticks from North-Western Spain. Ticks Tick-Borne Dis..

[27] Ebani V.V., Nardoni S., Mancianti F. (2023). Arthropod-Borne Pathogens in Wild Canids. Vet. Sci..

[28] Hrazdilová K., Lesiczka P.M., Bardoň J., Vyroubalová Š., Šimek B., Zurek L., Modrý D. (2021). Wild boar as a potential reservoir of zoonotic tick-borne pathogens. Ticks Tick-Borne Dis..

[29] Spitzer R. (2019). Trophic Resource Use and Partitioning in Multispecies Ungulate Communities. Ph.D. Thesis.

[30] Jaenson T.G., Jaenson D.G., Eisen L., Petersson E., Lindgren E. (2012). Changes in the geographical distribution and abundance of the tick Ixodes ricinus during the past 30 years in Sweden. Parasites Vectors.

[31] Accorsi A., Schiavetti I., Listorti V., Dellepiane M., Masotti C., Ercolini C., Guardone L., Razzuoli E. (2022). Hard Ticks (Ixodidae) from Wildlife in Liguria, Northwest Italy: Tick Species Diversity and Tick-Host Associations. Insects.

[32] Barker S.C., Murrell A. (2004). Systematics and evolution of ticks with a list of valid genus and species names. Parasitology.

[33] Estrada-Peña A., Bouattour A., Camicas J.L., Walker A.R. (2004). Ticks of Domestic Animals in the Mediterranean Region: A Guide to Identification of Species.

[34] Nava S., Beati L., Venzal J.M., Labruna M.B., Szabó M.P., Petney T., Saracho-Bottero M.N., Tarragona E.L., Dantas-Torres F., Silva M.M.S. (2018). *Rhipicephalus sanguineus* (Latreille, 1806): Neotype designation, morphological re-description of all parasitic stages and molecular characterization. Ticks Tick-Borne Dis..

[35] Schwaiger M., Cassinotti P. (2003). Development of a quantitative real-time RT-PCR assay with internal control for the laboratory detection of tick-borne encephalitis virus (TBEV) RNA. J. Clin. Virol..

[36] Stuen S., Nevland S., Moum T. (2003). Fatal cases of Tick-borne fever (TBF) in sheep caused by several 16S rRNA gene variants of *Anaplasma phagocytophilum*. Ann. N. Y. Acad. Sci..

[37] Skotarczak B., Wodecka B., Cichocka A. (2002). Coexistence DNA of *Borrelia burgdorferi* sensu lato and *Babesia microti* in *Ixodes ricinus* ticks from north-western Poland. Ann. Agric. Environ. Med..

[38] Berri M., Laroucau K., Rodolakis A. (2000). The detection of *Coxiella burnetii* from ovine genital swabs, milk and fecal samples by the use of a single touchdown polymerase chain reaction. Vet. Microbiol..

[39] Regnery R.L., Spruill C.L., Plikaytis B. (1991). Genotypic identification of rickettsiae and estimation of intraspecies sequence divergence for portions of two rickettsial genes. J. Bacteriol..

[40] Oteo J.A., Portillo A., Santibáñez S., Blanco J.R., Pérez-Martínez L., Ibarra V. (2006). Cluster of cases of human *Rickettsia felis* infection from Southern Europe (Spain) diagnosed by PCR. J. Clin. Microbiol..

[41] Choi Y.J., Jang W.J., Kim J.H., Ryu J.S., Lee S.H., Park K.H., Paik H.S., Koh Y.S., Choi M.S., Kim I.S. (2005). Spotted fever group and typhus group rickettsioses in humans, South Korea. Emerg. Infect. Dis..

[42] Richter J.R., Kimsey P.J., Madigan R.B., Barlough J.E., Dumler J.E., Brooks D.L. (1996). *Ixodes pacificus* (Acari: Ixodidae) as a vector of *Ehrlichia equi* (Rickettsiales: Ehrlichieae). J. Med. Entomol..

[43] Schets F.M., de Heer L., de Roda Husman A.M. (2013). *Coxiella burnetii* in sewage water at sewage water treatment plants in a Q fever epidemic area. Int. J. Hyg. Environ. Health.

[44] Briciu V.T., Sebah D., Coroiu G., Lupşe M., Cârstina D., Ţăţulescu D.F., Mihalca A.D., Gherman C.M., Leucuţa D., Meyer F. (2016). Immunohistochemistry and real-time PCR as diagnostic tools for detection of *Borrelia burgdorferi sensu lato* in ticks collected from humans. Exp. Appl. Acarol..

[45] Ivacic L., Reed K.D., Mitchell P.D., Ghebranious N. (2007). A Light Cycler TaqMan assay for detection of *Borrelia burgdorferi sensu lato* in clinical samples. Diagn. Microbiol. Infect. Dis..

[46] Hofmeester T.R., Coipan E.C., van Wieren S.E., Prins H.H.T., Takken W., Sprong H. (2016). Few vertebrate species dominate the *Borrelia burgdorferi s.l.* life cycle. Environ. Res. Lett..

[47] Fabri N.D., Sprong H., Hofmeester T.R., Heesterbeek H., Donnars B.F., Widemo F., Ecke F., Cromsigt J.P.G.M. (2021). Wild ungulate species differ in their contribution to the transmission of *Ixodes ricinus*-borne pathogens. Parasites Vectors.

[48] Matei I.A., Kalmár Z., Balea A., Mihaiu M., Sándor A.D., Cocian A., Crăciun S., Bouari C., Briciu V.T., Fiț N. (2023). The Role of Wild Boars in the Circulation of Tick-Borne Pathogens: The First Evidence of *Rickettsia monacensis* Presence. Animals.

[49] Pascucci I., Antognini E., Canonico C., Montalbano M.G., Necci A., di Donato A., Moriconi M., Morandi B., Morganti G., Crotti S. (2021). One health approach to rickettsiosis: A five-year study on spotted fever group rickettsiae in ticks collected from humans, animals and environment. Microorganisms.

[50] Selmi M., Ballardini M., Salvato L., Ricci E. (2017). *Rickettsia* spp. in *Dermacentor marginatus* ticks: Analysis of the host-vector-pathogen interactions in a northern Mediterranean area. Exp. Appl. Acarol..

[51] Petney T.N., Pfaeffle M.P., Skuballa J.D. (2012). An annotated checklist of the ticks (Acari: Ixodida) of Germany. Syst Appl. Acarol..

[52] Sevestre J., Diarra A.Z., Oumarou H.A., Durant J., Delaunay P., Parola P. (2021). Detection of emerging tick-borne disease agents in the Alpes-Maritimes region, southeastern France. Ticks Tick-Borne Dis..

[53] Mysterud A., Hügli C., Viljugrein H. (2021). Tick infestation on medium–large-sized mammalian hosts: Are all equally suitable to Ixodes ricinus adults?. Parasites Vectors.

[54] Perez G., Bournez L., Boulanger N., Fite J., Livoreil B., McCoy K.D., Quillery E., René-Martellet M., Bonnet S.I. (2023). The distribution, phenology, host range and pathogen prevalence of *Ixodes ricinus* in France: A systematic map and narrative review. Peer Community J..

[55] Bertola M., Montarsi F., Obber F., Da Rold G., Carlin S., Toniolo F., Porcellato E., Falcaro C., Mondardini V., Ormelli S. (2021). Occurrence and Identification of *Ixodes ricinus* Borne Pathogens in Northeastern Italy. Pathogens.

[56] Melis S., Batisti Biffignandi G., Olivieri E., Galon C., Vicari N., Prati P., Moutailler S., Sassera D., Castelli M. (2024). High-throughput screening of pathogens in *Ixodes ricinus* removed from hosts in Lombardy, northern Italy. Ticks Tick Borne Dis..

[57] Jore S., Vanwambeke S.O., Viljugrein H., Isaksen K., Kristoffersen A.B., Woldehiwet Z., Johansen B., Brun E., Brun-Hansen H., Westermann S. (2014). Climate and environmental change drives *Ixodes ricinus* geographical expansion at the northern range margin. Parasites Vectors.

[58] Medlock J.M., Hansford K.M., Bormane A., Derdakova M., Estrada-Peña A., George J.C., Golovljova I., Jaenson T.G., Jensen J.K., Jensen P.M. (2013). Driving forces for changes in geographical distribution of *Ixodes ricinus* ticks in Europe. Parasit Vectors.

[59] Sgroi G., Iatta R., Lia R.P., D’Alessio N., Manoj RR S., Veneziano V., Otranto D. (2021). Spotted fever group rickettsiae in *Dermacentor marginatus* from wild boars in Italy. Transbound. Emerg. Dis..

[60] Noll M., Wall R., Makepeace B.L., Newbury H., Adaszek L., Bødker R., Estrada-Peña A., Guillot J., Pereira da Fonseca I., Probst J. (2023). Predicting the distribution of *Ixodes ricinus* and *Dermacent* or reticulatus in Europe: A comparison of climate niche modelling approaches. Parasites Vectors.

[61] ECDC (2023). Ixodes Ricinus—Current Known Distribution. https://www.ecdc.europa.eu/en/publications-data/ixodes-ricinus-current-known-distribution-february-2023.

[62] Gandy S.L., Hansford K.M., Medlock J.M. (2023). Possible expansion of *Ixodes ricinus* in the United Kingdom identified through the Tick Surveillance Scheme between 2013 and 2020. Med. Vet. Entomol..

[63] Tagliapietra V., Rosà R., Arnoldi D., Cagnacci F., Capelli G., Montarsi F., Hauffe H.C., Rizzoli A. (2011). Saturation deficit and deer density affect questing activity and local abundance of *Ixodes ricinus* (Acari, Ixodidae) in Italy. Vet. Parasitol..

[64] Rizzoli A., Silaghi C., Obiegala A., Rudolf I., Hubálek Z., Földvári G., Plantard O., Vayssier-Taussat M., Bonnet S., Spitalská E. (2014). Ixodes ricinus and its transmitted pathogens in urban and peri-urban areas in Europe: New hazards and relevance for public health. Front. Public Health.

[65] Hansford K.M., Wheeler B.W., Tschirren B., Medlock J.M. (2022). Questing *Ixodes ricinus* ticks and *Borrelia* spp. in urban green space across Europe: A review. Zoonoses Public Health.

[66] Ceballos L.A., Pintore M.D., Tomassone L., Pautasso A., Bisanzio D., Mignone W., Casalone C., Mannelli A. (2014). Habitat and occurrence of ixodid ticks in the Liguria region, northwest Italy. Exp. Appl. Acarol..

[67] Ortuño A., Quesada M., López-Claessens S., Castellà J., Sanfeliu I., Antón E., Segura-Porta F. (2007). The role of wild boar (*Sus scrofa*) in the eco-epidemiology of *R. slovaca* in northeastern Spain. Vector-Borne Zoonotic Dis..

[68] Grech-Angelini S., Stachurski F., Lancelot R., Boissier J., Allienne J.F., Marco S., Maestrini O., Uilenberg G. (2016). Ticks (Acari: Ixodidae) infesting cattle and some other domestic and wild hosts on the French Mediterranean island of Corsica. Parasit Vectors.

[69] Dantas-Torres F. (2010). Biology and ecology of the brown dog tick, *Rhipicephalus sanguineus*. Parasit Vectors.

[70] Gray J., Dantas-Torres F., Estrada-Peña A., Levin M. (2013). Systematics and ecology of the brown dog tick, *Rhipicephalus sanguineus*. Ticks Tick Borne Dis..

[71] Curioni V., Cerquetella S., Scuppa P., Pasqualini L., Beninati T., Favia G. (2004). Lyme disease and babesiosis: Preliminary findings on the transmission risk in highly frequented areas of the Monti Sibillini National Park (Central Italy). Vector-Borne Zoonotic Dis..

[72] Marco I., Lopez-Olvera J.R., Rosell R., Vidal E., Hurtado A., Juste R., Pumarola M., Lavin S. (2007). Severe outbreak of disease in the southern chamois (*Rupicapra pyrenaica*) associated with border disease virus infection. Vet. Microbiol..

[73] Habib J., Zenner L., Garel M., Mercier A., Poirel M.T., Itty C., Appolinaire J., Amblard T., Benedetti P., Sanchis F. (2024). Prevalence of tick-borne pathogens in ticks collected from the wild mountain ungulates mouflon and chamois in 4 regions of France. Parasite.

[74] Ebani V.V., Bertelloni F., Turchi B., Filogari D., Cerri D. (2015). Molecular survey of tick-borne pathogens in Ixodid ticks collected from hunted wild animals in Tuscany, Italy. Asian Pacific J. Trop. Med..

[75] Pascucci I., Di Domenico M., Curini V., Cocco A., Averaimo D., D’Alterio N., Cammà C. (2019). Diversity of *Rickettsia* in Ticks Collected in Abruzzi and Molise Regions (Central Italy). Microorganisms.

[76] Chisu V., Foxi C., Masala G. (2018). First molecular detection of the human pathogen *Rickettsia raoultii* and other spotted fever group rickettsiae in Ixodid ticks from wild and domestic mammals. Parasitol. Res..

[77] Remesar S., Cano-Terriza D., Morrondo P., Jiménez-Ruiz S., López C.M., Jiménez-Martín D., Díaz P., Paniagua J., García-Bocanegra I. (2023). Molecular detection of *Rickettsia* spp. in wild ungulates and their ticks in Mediterranean areas of southwestern Spain. Zoonoses Public Health.

[78] Michalski M.M., Kubiak K., Szczotko M., Dmitryjuk M. (2021). Tick-Borne Pathogens in Ticks Collected from Wild Ungulates in North-Eastern Poland. Pathogens.

[79] Mancini F., Ciccozzi M., Lo Presti A., Cella E., Giovanetti M., Di Luca M., Toma L., Bianchi R., Khoury C., Rezza G. (2015). Characterization of spotted fever group *Rickettsiae* in ticks from a city park of Rome, Italy. Ann. Ist. Super Sanita.

[80] Gomez-Barroso D., Vescio M.F., Bella A., Ciervo A., Busani L., Rizzo C., Rezza G., Pezzotti P. (2019). Mediterranean spotted fever rickettsiosis in Italy, 2001–2015: Spatio-temporal distribution based on hospitalization records. Ticks Tick-Borne Dis..

[81] Grassi L., Menandro M.L., Cassini R., Mondin A., Pasotto D., Grillini M., Rocca G., Drigo M. (2022). High Prevalence of tick-borne zoonotic *Rickettsia slovaca* in ticks from wild boars, northeastern Italy. Animals.

[82] Barbiero A., Manciulli T., Spinicci M., Vellere I., Colao M.G., Rossolini G.M., Bartoloni A., Raoult D., Zammarchi L. (2023). Scalp eschar and neck lymph adenopathy after a tick bite (SENLAT) in Tuscany, Italy (2015–2022). Infection.

[83] Guccione C., Rubino R., Colomba C., Anastasia A., Caputo V., Iaria C., Cascio A. (2022). Rickettsiosis with Pleural Effusion: A Systematic Review with a Focus on Rickettsiosis in Italy. Trop. Med. Infect. Dis..

[84] ECDC—Epidemiological Situation of Rickettsioses in EU/EFTA Countries. https://op.europa.eu/en/publication-detail/-/publication/afe46de4-e6fb-4c38-9661-d2ce0d3b05b0/language-en.

[85] Zhang Y.Y., Hay S.I., Fang L.Q., Sun Y., Chen J., Teng A., Wang T., Li H., Hay S.I., Fang L. (2023). Mapping the global distribution of spotted fever group rickettsiae: A systematic review with modelling analysis. Lancet Digit. Health.

[86] Grassi L., Drigo M., Cassini R., Mondin A., Pasotto D., Sinigaglia R., Rocca G., Menandro M.L. (2022). High Prevalence of *Rickettsia Slovaca* in *Dermacentor Marginatus* in Euganean Hills Regional Park. Int. J. Infect. Dis..

[87] Boldis V., Spitalská E. (2010). *Dermacentor marginatus* and *Ixodes ricinus* ticks versus L929 and Vero cell lines in *Rickettsia slovaca* life cycle evaluated by quantitative real time PCR. Exp. Appl. Acarol..

[88] Raele D.A., Galante D., Pugliese N., Salandra G., Cafiero M.A. (2018). Spotted fever group rickettsiae associated with ixodid ticks in wild environment in Southern Italy. Microbiol. Open.

[89] Garcia-Vozmediano A., Giglio G., Ramassa E., Nobili F., Rossi L., Tomassone L. (2020). *Dermacentor marginatus* and *Dermacentor reticulatus*, and their infection by SFG *Rickettsiae* and *Francisella*-like endosymbionts, in mountain and periurban habitats of Northwestern Italy. Vet. Sci..

[90] Blanda V., Torina A., la Russa F., D’Agostino R., Randazzo K., Scimeca S., Giudice E., Caracappa S., Cascio A., de la Fuente J. (2017). A Retrospective Study of the Characterization of *Rickettsia* Species in Ticks Collected from Humans. Ticks Tick-Borne Dis..

[91] Parola P., Paddock C.D., Socolovschi C., Labruna M.B., Mediannikov O., Kernif T., Abdad M.Y., Stenos J., Bitam I., Fournier P. (2013). Update on tick-borne rickettsioses around the world: A geographic approach. Clin. Microbiol. Rev..

[92] Karbowiak G., Biernat B., Stańczak J., Szewczyk T., Werszko J. (2016). The role of particular tick developmental stages in the circulation of tick-borne pathogens affecting humans in Central Europe. 3. Rickettsiae. Ann. Parasitol..

[93] Madeddu G., Mancini F., Caddeo A., Ciervo A., Babudieri S., Maida I., Fiori M.L., Rezza G., Mura M.S. (2012). *Rickettsia monacensis* as cause of Mediterranean spotted fever-like illness, Italy. Emerg. Infect. Dis..

[94] Maioli G., Pistone D., Bonilauri P., Pajoro M., Barbieri I., Mulatti P., Vicari N., Dottori M. (2012). Etiological agents of rickettsiosis and anaplasmosis in ticks collected in Emilia-Romagna region (Italy) during 2008 and 2009. Exp. Appl. Acarol..

[95] Scarpulla M., Barlozzari G., Marcario A., Salvato L., Blanda V., De Liberato C., D’Agostini C., Torina A., Macrì G. (2016). Molecular detection and characterization of spotted fever group rickettsiae in ticks from Central Italy. Ticks Tick Borne Dis..

[96] Cascio A., Torina A., Valenzise M., Blanda V., Camarda N., Bombaci S., Iaria C., De Luca F., Wasniewska M. (2013). Scalp eschar and neck lymphadenopathy caused by *Rickettsia massiliae*. Emerg. Infect. Dis..

[97] Mediannikov O., Matsumoto K., Samoylenko I., Drancourt M., Roux V., Rydkina E., Davoust B., Tarasevich I., Brouqui P., Fournier P.E. (2008). *Rickettsia raoultii* sp. nov., a spotted fever group rickettsia associated with *Dermacentor* ticks in Europe and Russia. Int. J. Syst. Evolut. Microbiol..

[98] Chochlakis D., Ioannou I., Tselentis Y., Psaroulaki A. (2010). Human anaplasmosis and *Anaplasma ovis* variant. Emerg. Infect. Dis..

[99] Li H., Zheng Y.C., Ma L., Jia N., Jiang B.G., Jiang R.R., Huo Q.B., Wang Y.W., Liu H.B., Chu Y.L. (2015). Human infection with a novel tick-borne *Anaplasma* species in China: A surveillance study. Lancet Infect. Dis..

[100] Lu M., Chen Q., Qin X., Lyu Y., Teng Z., Li K., Yu L., Jin X., Chang H., Wang W. (2022). *Anaplasma bovis* infection in fever and thrombocytopenia patients—Anhui Province, China, 2021. China CDC Wkly..

[101] Lu M., Li F., Liao Y., Shen J.J., Xu J.M., Chen Y.Z., Li J.H., Holmes E.C., Zhang Y.Z. (2019). Epidemiology and diversity of rickettsiales bacteria in humans and animals in Jiangsu and Jiangxi provinces, China. Sci. Rep..

[102] Da Rold G., Obber F., Monne I., Milani A., Ravagnan S., Toniolo F., Sgubin S., Zamperin G., Foiani G., Vascellari M. (2022). Clinical tick-borne encephalitis in a roe deer (*Capreolus capreolus* L.). Viruses.

[103] Pacilly F.C.A., Benning M.E., Jacobs F., Leidekker J., Sprong H., Van Wieren S.E., Takken W. (2014). Blood feeding on large grazers affects the transmission of *Borrelia burgdorferi sensu lato* by *Ixodes ricinus*. Ticks Tick-Borne Dis..

[104] Di Domenico M., Pascucci I., Curini V., Cocco A., Dall’Acqua F., Pompilii C., Cammà C. (2016). Detection of *Anaplasma phagocytophilum* genotypes that are potentially virulent for human in wild ruminants and *Ixodes ricinus* in central Italy. Ticks Tick-Borne Dis..

